# Using an Interactive Systems Framework to Expand Telepsychology Innovations in Underserved Communities

**DOI:** 10.1155/2016/4818053

**Published:** 2016-06-15

**Authors:** Whitney R. Garney, Carly E. McCord, Michaela V. Walsh, Angela B. Alaniz

**Affiliations:** ^1^Department of Health & Kinesiology, Texas A&M University, College Station, TX 77843, USA; ^2^School of Public Health, Texas A&M Health Science Center, College Station, TX 77843, USA; ^3^Department of Educational Psychology, Texas A&M University, College Station, TX 77843, USA

## Abstract

Literature indicates that the use of promising innovations in mental health care can be improved. The advancement of telepsychology is one innovation that has been utilized as a method to reduce rural health disparities and increase the number of people with access to mental health services. This paper describes a successful pilot telepsychology program implemented in a rural community to increase access to mental health services and the model's replication and expansion into four additional communities using concepts described in an Interactive Systems Framework. The Interactive Systems Framework highlights how building local capacity specific to organizational functioning and innovations are necessary to support, deliver, and disseminate innovations within new settings. Based on the knowledge gained from this telepsychology innovation, the application of an Interactive Systems Framework and funding mechanisms are discussed.

## 1. Introduction

To meet the needs of an ever-changing environment of mental health, promising innovations in prevention, practice, treatment, and promotion are essential [1]. Broadly defined, innovations are new approaches, interventions, or strategies that are informed by scientific theory or empirical evidence [2, 3]. Promising innovations have undergone preliminary testing and have the potential to result in substantial change to current practice. The foremost theory on innovation is called Diffusion of Innovations [2]. In 1962 [4], Rogers published the first edition of his seminal book,* Diffusion of Innovations*, which drastically increased the popularity of diffusion research. Since then, researchers and practitioners have frequently used concepts outlined in the Diffusion of Innovation to disseminate and replicate new practices or procedures; one such adaptation is the Interactive Systems Framework, which is the focus of this paper [2, 5].

Literature indicates that the use of promising innovations in mental health care can be improved [6]. Published research shows that challenges in disseminating and adopting innovations can inhibit how the Diffusion of Innovation theory is applied in mental health [3, 7]. As a result, novelties in evidence-based treatment are not always adopted by practitioners. One such example is the use of telepsychology for service provision. Telepsychology is defined by the American Psychological Association as “the provision of psychological services using telecommunication technologies” [8]. These telecommunication technologies include telephone, mobile devices, videoconferencing, email, and chat, to name a few. Meta-analyses and systematic reviews have demonstrated the effectiveness of telehealth and widespread, growing acceptance from consumers and health care providers [9, 10]. The American Telemedicine Association has Guidelines for Video-Based Online Mental Health Services (2013) [11].

To date, no studies have identified a patient subgroup that does not benefit from or is harmed by mental health care provided through remote videoconferencing. Recent large randomized control trials demonstrate effectiveness of telemental health with many small trials also supporting this conclusion (p. 9).

Despite the growing empirical support for telepsychology, adoption of this service delivery modality remains slow as implementation of this innovation can be difficult. This paper demonstrates how an Interactive Systems Framework, which uses key Diffusion of Innovation principals, can be applied in mental health to expand telepsychology services to an underserved area. First, we describe and illustrate the Interactive Systems Framework (ISF) theory used in our conceptualization of this project. Then, we detail the pilot project, which used an innovation in telepsychology and a university training program to meet the needs of underserved, rural residents and can be understood via the ISF theory.

## 2. The Interactive Systems Framework

**Figure 1:**
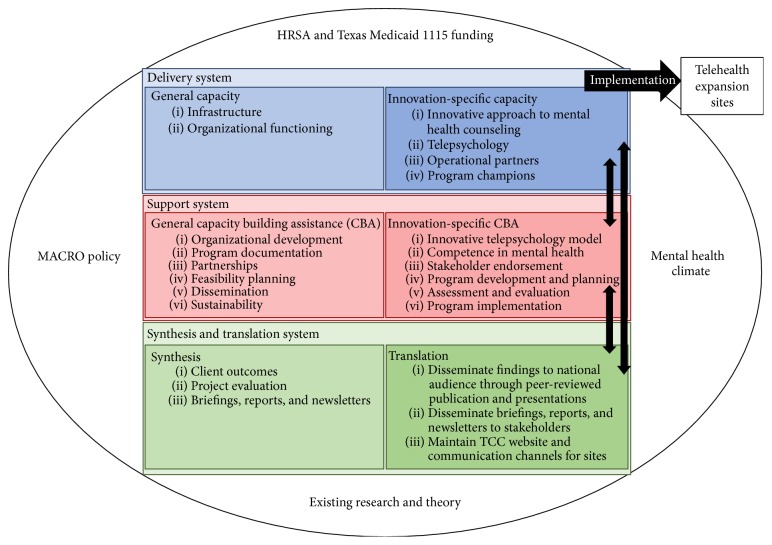
Interactive system framework model.

The Interactive Systems Framework (ISF) is an approach originally developed by Abraham Wandersman to disseminate innovations into new settings [12]. Its purpose is to distill information generated through pilot projects and research and prepare it for dissemination and adoption in the field. The ISF has three main components: (1) Support System, (2) Delivery System, and (3) Synthesis and Translation System. Bidirectional arrows connect the three components, which allows for feedback among the systems and culminates in innovations being expanded into different settings [12]. The framework is intended to be used by practitioners, researchers, and other stakeholders to translate promising innovations into new settings using a multidisciplinary perspective [13]. A conceptual model of the ISF, utilized for the mental health project described in this paper, is available in Figure 1 [12].

### 2.1. Delivery System

The purpose of the ISF Delivery System is to help* carry out activities* necessary to replicate and adopt innovations. One function of this system is to stimulate an environment of innovation for program staff that will enhance organizational functioning [4]. The shared culture of innovation promotes common values and behaviors that support an innovative philosophy, thus altering norms to support creativity and novel thinking [14]. The ISF assists in developing partnerships with stakeholders relevant to the innovative project, including the intended target population and community supporters. These partnerships both inform potential program modifications and add to the potential for dissemination and successful adoption.

### 2.2. Support System

The ISF Support System builds capacity to carry out program activities (within the Delivery System) through supportive functions like training and technical assistance [15]. Innovators operationalize the general capacity (specifically related to organizational functioning) and innovation-specific capacity (specifically related to program innovation) which they develop to accentuate their programs, leverage resources, and enhance other programmatic aspects of activities.

General capacity is necessary to adapt an innovation to a new setting. It is developed through capacity building assistance (CBA) focused on organizational functioning. CBA enhances organizational infrastructure and support, as well as skills in planning, monitoring, dissemination, and sustainability [13]. CBA activities are generally provided by a third party that has experience in the innovation and can help new adapters implement the program. Innovation-specific CBA focuses on the program-related aspects of innovations and aims to build capacity necessary to carry out new activities. These activities can range from developing protocols and procedures that help staff implement a new program to developing technological expertise to carry out various aspects of the innovation.

### 2.3. Synthesis and Translation System

Following the implementation of a new innovation, new information is generated. This information can be internal (e.g., related to how well the innovation fits within the organization) or external (e.g., outside evaluation). The ISF Synthesis and Translation System takes this information and prepares it for dissemination. Synthesis and translation activities are typically carried out by project staff and then disseminated to stakeholders through project reports, presentations, publications, and newsletters.

The following section describes how the ISF can be used to disseminate mental health innovations into new settings. First, we use the Leon County pilot project as an example of how an innovation can be utilized in new settings by developing capacity building at local levels, focusing on community ownership, and embedding practices into organizational functions.

Then, we illustrate how the telepsychology pilot project was replicated and carried out in relation to the components of ISF.

## 3. Leon County: Adopting Innovation

In 2006, Leon County, a Mental Health Professional Shortage Area in the Brazos Valley region of East Central Texas, partnered with the Center for Community Health Development (CCHD) at the Texas A&M (TAMU) Health Science Center, School of Public Health, to develop a community-based strategy to address lack of access to mental health services. The partnership reached out to the Counseling and Assessment Clinic, a community-based clinic housed at the TAMU Department of Educational Psychology, to develop and implement a telepsychology pilot project. This was done by involving Educational Psychology doctoral students to deliver free services to rural residents. In 2007, the Health Resources and Services Administration Office of Rural Health Policy funded Leon County, CCHD, and the CAC to test the innovation. With nearly a quarter of Leon County residents being uninsured, counseling services needed to be available at a free or reduced cost. As a psychological service training clinic, the CAC counseling services were provided at no cost by faculty-supervised doctoral students from the Texas A&M Counseling Psychology Program, making the student-based service delivery model an optimal fit for the pilot project. The partnership between Leon County and CAC proved beneficial to both entities, as CAC doctoral students received valuable clinical training utilizing a new method of service delivery and Leon County residents were able to obtain free mental health services [16, 17].

### 3.1. Telepsychology Model Infrastructure

The Leon Health Resource Center (HRC), a county-owned and operated facility, provided space for the telepsychology services. The Leon HRC staff acted as the first point of contact for clients when they arrived for counseling. The staff also provided administrative support for the telepsychology program, faxing client paperwork to CAC counselors and interfacing with counselors as needed. Connectivity for the telepsychology services were established using a T-1 line that securely connected Leon County to the Texas A&M network infrastructure. This line was compliant with Health Insurance Portability and Accountability Act (HIPAA) regulations.

### 3.2. Service Delivery

Leon County residents accessed the telepsychology services locally at the Leon HRC, while counselors connected in remotely from offices at TAMU. The distance between the sites is over an hour away, which illustrates the importance of mitigating distance in terms of administering mental health to underserved populations. In many ways, the counseling provided via telepsychology was almost identical to in-person counseling services. Clients were scheduled for weekly 50-minute sessions and seen by a trained, advanced Educational Psychology doctoral student. Student clinicians used a variety of theoretical orientations and interventions based on client needs including cognitive behavioral therapy, cognitive processing therapy, solution-focused therapy, problem-solving training, prolonged exposure therapy, and humanistic approaches.

### 3.3. Results

Prior to implementation, very limited mental health services were available within the county. As a result of the telepsychology project, 168 residents have received psychological services via telepsychology from 2009 to 2015. Considering the fact that these clients would not normally be able to access mental health services, the outcomes of the project are substantial. Not only do clients demonstrate statistically significant decreases in symptoms after even four sessions of counseling, but clinically significant changes in client's productivity and satisfaction with life are readily observable [18, 19]. Anecdotally, as new coping skills are learned, clients also report reductions in emergency service utilization thereby creating significant cost savings for clients, hospitals, and communities.

Clients were seen an average of 8.13 (SD = 10.7) sessions and a total of 1366 encounters. The number of sessions per client ranged from 1 to 61 with an average of 1.91 (SD = 2.68) no-shows. Clients were predominately female (75.0%) and Caucasian (75.6%). Other ethnicities represented include African American (8.9%), Hispanic (12.5%), Biracial (2.4%), Asian (.6%), and “Other” (2.0%). Clients range in age from 9 to 73 years old with an average age of 40.32 (SD = 14.1). The most common diagnoses have been depression, anxiety, relationship concerns, and traumatic stress.

### 3.4. Sustainability

The Leon County pilot project was designed to ensure long-term sustainability by leveraging resources to minimize the cost of care without sacrificing quality. Much of the infrastructure required in Leon County was already supported by the county government, like the space provided at the Leon HRC. Therefore, the cost of the T-1 line, approximately $6,000 annually, was the only additional expense the county had to absorb to continue the services and the Leon HRC staff has assumed the local telepsychology-related responsibilities. As a result of this pilot and other opportunities later described, CAC faculty and CCHD established the Telehealth Counseling Clinic (TCC), which operates under the Texas A&M Health Science Center, to deliver telepsychology services.

### 3.5. Dissemination

The results of the project have been disseminated to the scientific community through peer-reviewed publications [16, 18, 19] and the Leon HRC staff has presented findings at community meetings throughout the region. The TCC has also shared results with rural leaders and health care providers across the Brazos Valley region, which led to opportunities to replicate the model in other rural areas. While much of the replication process happened organically, CCHD and TCC focused on building local capacity to deliver and sustain the telepsychology services once implemented. This capacity was delivered using a participatory approach and is described using an Interactive Systems Framework (ISF) as described in the previous section. Overall, five remote telepsychology sites have been established in the Brazos Valley region. Over 500 clients have been served for a total of almost 5,000 encounters. Clients are primarily rural residents who would not normally receive mental health services locally; thus the program has drastically increased access to services in the short period of time. Furthermore, preliminary analyses indicate significant decreases in PHQ depression scores (*t* = 6.61, *p* < .01) between the first session (M = 14.98, SD = 7.34) and fifth session (M = 10.92, SD = 6.66) with Cohen's *d* effect size of .56. The age range for clients is from 9 to 78 and the mean age was 41.13 (SD = 6.2).

## 4. Expanding Telepsychology in Other Underserved Areas: A Case Study in ISF

The following section describes how the ISF can be used to disseminate mental health innovations into new settings. We use the Leon County pilot project as an example of how an innovation can be utilized in new settings by developing capacity building at local levels, focusing on community ownership, and embedding practices into organizational functions.

### 4.1. Funding Mechanism

Two primary funding mechanisms were used to replicate the innovative telepsychology pilot project. In total, the pilot project was expanded into four additional Brazos Valley counties, with three communities using funds available from a state Medicaid 1115 waiver program and the other county using funding procured by a three-year HRSA grant.

### 4.2. Medicaid 1115 Waiver

In December 2011, the Centers for Medicare and Medicaid Services (CMS) granted a five-year 1115 Medicaid waiver to Texas which created a financial incentive program for health care providers who opted to implement changes aimed at improving care, increasing efficiency, and decreasing costs. While these changes benefit all patients, they also must prove to have a positive, quantifiable patient impact on Medicaid and low-income uninsured patients. The waiver's Delivery System Reform Incentive Program (DSRIP) allows eligible entities to develop projects and receive incentive payments from CMS. As part of the Texas A&M Health Science Center, an eligible DSRIP provider under the waiver, the TCC had the opportunity to expand its service delivery in the Leon County pilot project to three additional Brazos Valley counties (Brazos, Grimes, and Washington). With the majority of the TCC's patients being uninsured or covered by Medicaid, the TCC's patient population aligned with the waiver's target population.

### 4.3. Health Resource and Services Administration

HRSA works to improve access to health care for underserved and vulnerable populations, specifically those who are unable to access quality care due to geographic, economic, or medical limitations. Expanding mental health services to rural communities using telepsychology aligns well with HRSA's goals. As a result, in 2011, Madison County applied for an ORHP Service Expansion grant from HRSA and was awarded funding in 2012. The purpose of the grant was to replicate the telepsychology model pilot tested in Leon County and expand it to address comorbidities of mental health and substance abuse. The county worked with CCHD and TCC to carry out program activities once awarded the grant.

### 4.4. Interactive Systems Framework Delivery System 

To replicate the telepsychology innovation from Leon County in four new communities, CCHD and TCC focused first on developing local capacity. Two types of capacity are essential for adopting an innovation—general capacity and innovation-specific capacity.

#### 4.4.1. General Capacity

The first step to replication required community ownership and commitment to absorb the telepsychology program locally. Similar to Leon County, three of the four counties (Grimes, Madison, and Washington) housed the telepsychology services in local health resource centers. In Brazos County, TCC partnered with a nonprofit health clinic to deliver services. CCHD and TCC partnered with the county and/or city governments to adapt the telepsychology model for each community. This partnership process was essential because it ensured that communities were intimately involved in developing and implementing the project. It also identified stakeholders early on in the process who were engaged and would ultimately take ownership of the services once external funding ended. The types of community partners differed in each county based on organizational resources and local interest. Community partners ranged from the local county governments, critical access hospitals, local nonprofits, and Health Resource Centers.

Organizational procedures at TCC (the service provider) and local community sites also had to evolve to accommodate the telepsychology program. Three of the new sites used a locally installed T-1 line that connected to TCC through Texas A&M's network. At the fourth site, a business class, cable internet solution was used. TCC expanded its use of electronic records to adequately track patients through Titanium Schedule, a HIPAA-compliant system for scheduling, record keeping, billing, and data management.

#### 4.4.2. Innovation-Specific Capacity

To easily implement the mental health innovation at each community site, TCC established an administrative protocol that described who was responsible for greeting the clients, collecting necessary paperwork and assessment tools, and ensuring the telepsychology unit was turned on when sessions started. Remote site staff sign confidentiality agreements with the TCC and are required to complete training in HIPAA. The TCC staff work with the remote sites to customize the administrative processes, so they are seamlessly integrated into the facility's existing protocols.

The TCC also created specific tools for intake, treatment, referral, and handling emergencies during counseling sessions. Clients are screened initially over the phone to look for any signs that the person may not be a good fit for telepsychology including severity of symptoms or suspiciousness of technology. Clients who are severely suicidal or homicidal or have psychotic symptoms and are not medicated are not a good fit for solely long distance services. Often, clients seeking services with such severe symptomology are referred to services with more adequate levels of care and then are reevaluated for telepsychology services once they have been stabilized. During phone screenings, clients are informed of the relevant technology being used in telepsychological services as well as the security of their clinical data.

### 4.5. ISF Support System

CCHD and TCC conducted capacity building assistance with local organizations to enhance skills, knowledge, and resources needed to implement the telepsychology project. This assistance was used by local stakeholders to implement the innovation within the ISF's Delivery System previously described.

#### 4.5.1. General Capacity Building Assistance

To help local organizations in the four counties carry out activities necessary to implement the telepsychology project, CCHD helped develop the local infrastructure to accommodate new activities. CCHD worked with stakeholders to establish oversight mechanisms, project budgeting, and processes to disseminate results.

For example, in Madison County, local partners formed the Madison Outreach and Services through the Telehealth (MOST) Network. This group of providers met bimonthly to discuss the project and solve any issues related to services, connectivity, or client needs. These meetings provided an opportunity for partners to communicate about project activities and review progress to make sure that services were delivered effectively and efficiently.

CCHD and TCC also worked with the local HRCs to incorporate telepsychology into standard operating procedures performed by existing staff, so that the telepsychology services are incorporated into the daily activities of the center. Each HRC has an executive director who is responsible for overseeing the telepsychology services, as well as an office manager who coordinates the daily scheduling and paperwork for clients. CCHD assisted the HRC in budgeting for services and planning for sustainability once the seed funding from HRSA and the Medicaid 1115 waiver ends. All of these CBA activities are aimed at helping the organizations in absorbing the telepsychology services into their existing organization structure.

#### 4.5.2. Innovation-Specific Capacity Building Assistance

Prior to services starting at each remote site, TCC staff trained the site staff on policies and procedures including day-to-day operations and handling emergencies. In addition to the standard training, the TCC staff works with each site to maximize effectiveness and ensure that the unique sites' needs are met. The TCC's staff and counselors also assist remote sites in troubleshooting technical issues. Any technical issues not solved by the TCC counselor require an IT professional to be dispatched to the site, which could result in a site being unusable for days at a time.

The TCC also developed a training program geared at equipping the next generation of psychologists for delivering telepsychology services. This training program not only provides students with many of the functional and foundational competencies necessary for the provision of counseling services in any setting but also gives students unique training experiences through practice and didactic technology trainings. Trainees learn technical skills related to using technology for service delivery, troubleshooting problems, and interfacing with information technology professionals. Clinically, trainees learn how to assess appropriateness for telepsychology, how to make decisions about a client's care during a technology outage, and how to select and implement appropriate interventions via telepsychology. Readers interested in an in-depth look at the TCC's training program are referred to work published in 2015 by McCord et al. [20].

### 4.6. ISF Synthesis and Translation

#### 4.6.1. Synthesis

TCC routinely aggregates patient data and compiles reports on county-specific and overall service delivery. These reports are essential in both understanding the results of the program and communicating these findings to a larger audience. Local sites also collect utilization data which they synthesize and present to their stakeholders and community partners.

#### 4.6.2. Translation

The TCC uses weekly clinical assessments to track changes in symptoms, improvements in functioning, and well-being to assess client progress. They also use data for internal continuous quality improvements. Ultimately, TCC aims to develop and promote best practices for academic-community partnerships in telepsychology service delivery and training; therefore, they disseminate results to a large academic audience through psychology and community-based journals [20–23] as well as professional conferences. TCC also shares services numbers with project stakeholders through a monthly e-mail update.

Community sites review utilization reports internally among staff to prioritize resources and plan for future activities. They also include service numbers and results in annual reports which they present to local funders. These annual reports help the sites ensure that their operational funding continues and help attract new sources of funding.

## 5. Discussion

Telepsychology is one example of a mental health innovation used to improve the delivery of behavioral health services in rural settings [24]. The telepsychology innovation described in this paper has been implemented and tested, expanded, and sustained in five counties since 2007. Overall, this paper offers insight into how to leverage funding, how to develop infrastructure and service delivery processes necessary to adopt innovations on behalf of communities and practitioners, and how capacity building assistance is necessary to ensure sustainability of services and embed innovations into existing organizational processes.

This project utilizes the Interactive Systems Framework to describe how mental health innovations can be expanded within new practitioner and community settings. The model was designed to be used by multiple disciplines and is a valuable framework to understand how innovations are conceptualized, utilized, and disseminated to inform future work.

The ISF provides a structure for adapting an innovation to a particular situation with its emphasis on building and utilizing capacity at various levels. However, it does not necessarily help in navigating challenges that can occur in implementation. Practitioners involved in the expansion of telehealth have had difficulties in obtaining organizational/institutional support, overcoming technical challenges like connectivity in rural areas, and addressing transportation issues to get clients from their home to the local service provider. One of the most useful lessons learned from this project was the importance of human capital. In the cases outlined in this paper, practitioners learned that it takes people who are committed to the fulfillment of framework to make it viable. These individuals were not necessarily the mental health practitioners who were delivering the telepsychology services; instead, they were vested members of the community who wanted to increase access to services.

The outside individuals involved in implementation are not a strong focus within in the ISF. Therefore, a suggestion for future use of the framework is to include aspects of implementation of science research in the ISF's Prevention Support System. One specific resource that is relevant to the ISF is the Consolidated Framework for Implementation Research (CFIR), which is a guideline that describes characteristics of five domains related to implementing interventions [25]. These domains are (1) an intervention's characteristics, (2) the outer setting, (3) the inner setting, (4) characteristics of the individuals, and (5) process. By reframing “intervention” as “innovation,” CFIR highlights important considerations in the implementation process, which can be used to enhance the ISF. This includes capitalizing on contextual considerations like the community environment (outer setting) where an innovation is being implemented and examining characteristics of both the individuals responsible for implementation and outside stakeholders who can offer assistance or support in the implementation process.

### 5.1. Other Telepsychology Considerations

For continued expansion of innovations that reduce mental health care disparities, such as telepsychology, to be possible, several considerations related to funding, reimbursement laws, and interjurisdictional practice must be considered. Due to governmental support, federal funding sources like HRSA are ideal sources for start-up costs in innovative mental health projects. In addition, other federal funding streams like Texas' Medicare 1115A Waiver Program offer opportunities to establish infrastructure to deliver mental health care to uninsured residents. These funding sources assist public health and mental health professions overcome barriers like the high start-up costs of innovative programs.

The relatively low maintenance costs associated specifically with telepsychology services increase feasibility of local sustainability if community support is established early in the process. Community buy-in is essential to the success of the program and planning for local sustainability must be addressed prior to beginning telepsychology services. If there is no commitment to sustain the services after the seed funding has ended, providers should carefully consider the risk of initiating services.

Overall, potential funding sources exist for innovative mental health programs; however, they may not present as traditional funding mechanisms like National Institutes of Health grants or other such mechanisms. As in this project, nontraditional sources of funding should be pursued when innovations are both needed and feasible in underserved populations. In community-based mental health services specifically, such as the one described in this paper, local community support was essential in sustaining programs once start-up funds ended. This step should not be overlooked.

Although federal and state support for telepsychology is evident, policy changes are needed to promote telepsychology innovations in the current health care environment. Changes in policies related to billing, reimbursement, provider restrictions, and provider training should be considered. The current policies regarding billing and reimbursement for telepsychology services are vague and vary from state to state. In Texas, medical providers can bill public insurers and the physician receives reimbursement for services, but there must be a licensed nurse on the patient end of the connection; furthermore, the nurse can only bill for a fraction of the service. This creates a disincentive for the receiving end of the services, especially if they are contracting the providing end for care. Most providers hesitate because of the increased administrative burden and the low reimbursement. Private insurers' approach to telepsychology services also varies greatly. Comprehensive and straightforward policies should define telepsychology services in a standard way, and there should be some consistency in how services are billed and reimbursed across payers and states.

The Association of the State and Provincial Psychology Boards (ASPPB) is propelling the field forward by writing the Psychology Interjurisdictional Compact also known as PSYPACT. The purpose of the compact is to facilitate telepsychology and in-person psychology practices across jurisdictional boundaries. An interstate compact, like the name implies, is a contract between states. It serves a dual role as both a statute and a contract such that, once the compact is ratified by a state, the provisions set forth by the compact will take precedent over the current state laws. Having states ratify PSYPACT would be a huge leap forward in advancing telepsychology practices across the nation thereby making an impact on mental health disparities.
